# Loot boxes are again linked to problem gambling: Results of a replication study

**DOI:** 10.1371/journal.pone.0213194

**Published:** 2019-03-07

**Authors:** David Zendle, Paul Cairns

**Affiliations:** 1 Department of Computer Science, York St. John University, York, North Yorkshire, United Kingdom; 2 Department of Computer Science, University of York, York, North Yorkshire, United Kingdom; University of Auckland, NEW ZEALAND

## Abstract

Loot boxes are items in video games that contain randomised contents and can be purchased with real-world money. Similarities between loot boxes and forms of gambling have led to questions about their legal status, and whether they should be regulated as gambling. Previous research has suggested a link between the amount that gamers spend on loot boxes and their problem gambling: The more individuals spent on loot boxes, the more severe their problem gambling. However, the generalisability of prior work may be limited by both the self-selected nature of the sample under test, and the fact that participants were aware of the study’s aims. A large-scale survey of gamers (n = 1,172) was undertaken to determine if this link remained when these limitations of previous work were taken into account. These gamers did not self-select into a loot box study and were not aware of the study’s aims. This study found similar evidence for a link (η^2^ = 0.051) between the amount that gamers spent on loot boxes and the severity of their problem gambling. Previous research strongly suggested both the size and the direction of link between loot box use and problem gambling. This paper provides further support for this link. These results suggest either that loot boxes act as a gateway to problem gambling, or that individuals with gambling problems are drawn to spend more on loot boxes. In either case, we believe that these results suggest there is good reason to regulate loot boxes.

## Introduction

Loot boxes are a profitable mechanism in modern video games that shares significant psychological and structural similarities with gambling. There is concern that loot boxes may pose risks to gamers, and some territories have already regulated them as a form of gambling. Previous research has suggested the existence of an important link between loot box spending and problem gambling. However, the self-selected and unblinded nature of the sample used in this research limits its generalisability. In the research outlined below we replicate the existence of this link with a sample where individuals are not aware of the survey’s aims.

### Loot boxes are big business

Loot boxes are items in video games that players can buy with real-world money, but which, when opened, contain randomised contents. They are very widespread in modern video games, and a recent report by the UK Gambling Commission estimated that 31% of children aged 11–16 have opened one[1]. Loot boxes are highly profitable, with some estimates stating that they will generate up to $30 billion dollars for the video game industry in 2018 alone[2].

#### Loot boxes share psychological and structural features with gambling

Loot boxes all share one common feature: When players purchase one, they do not know what specific thing they will receive in return for their money. For example, players of the first-person shooter *Counter-Strike*: *Global Offensive* can pay real-world money to unlock sealed weapon cases, which have randomised contents. Players do not know what a weapon case’s contents are when they pay to unlock it. It might contain items that are both rare and valuable: For example, a case might contain the *Dragon Lore* gun skin[3], which carries enormous value on secondary markets–indeed, it can be resold to other players for over $4000[4]. On the other hand, a weapon case might contain an ugly or common item that is of little or no value.

The chance-based nature of loot boxes has led to questions over similarities between them and gambling. Gambling experts have noted that loot boxes share several key formal features with traditional forms of gambling. For example, both when wagering at the roulette wheel, and when buying a loot box, players are risking the loss of real-world money for the chance of obtaining a valuable reward [5].

These structural similarities have led to questions over whether loot boxes might pose similar risks to gambling. More specifically, there is concern that spending on loot boxes might form a gateway to problem gambling amongst gamers. Problem gambling is an excessive and involuntary pattern of gambling activity which causes serious problems in an individual’s personal, family, and vocational life [6]. It is thought to often be caused by conditioning from arousing features of gambling [7]. Drummond and Sauer[8] analysed 22 games featuring loot boxes to determine if they shared the characteristics of gambling necessary for them to lead to the development of problem gambling amongst gamers. They concluded that many loot boxes shared “important structural and psychological similarities with gambling” and recommended their regulation lest they create a “ripe breeding ground” for problem gambling.

### Some bodies have concluded that loot boxes should be regulated as gambling

Indeed, some regulators have formally investigated whether loot boxes share enough similarities with forms of gambling, and determined that they should legally be regulated as gambling themselves. Early in 2018 Belgian and Dutch authorities ruled that some loot boxes violated national gambling legislation, and ordered that they be removed from video games. [9], [10]

This hard stance on loot box regulation is, however, far from universal. A recent Australian Senate investigation into loot boxes did not declare them illegal as a form of gambling, but instead ordered that a “comprehensive review” of national gambling legislation should take place in order to ensure that it was still current [11]. French gambling authorities have ruled that because loot box items lack direct real-world monetary value, purchasing them cannot be classified as gambling [12]. Similarly, the UK Gambling Commission determined that loot boxes do not legally constitute gambling because “the prizes unlocked in loot boxes are usable only in the games in which they’re won.”[13]. It is important to note that this perspective on loot boxes does not take into account well-known secondary markets such as *OPSkins*[14], on which loot box winnings are regularly ‘cashed out’ by gamers for real-world money through a resale process. This is a common feature of many games that feature loot boxes [8]. Further information on the different approaches to legislating loot boxes are available in [5], [15], [16].

### Recent data suggests an important link between loot box spending and problem gambling

Criticism of loot boxes has been roundly rebuffed by the games industry. Loot boxes do not represent the first time that consumers have been given randomised rewards in return for spending real-world money. Indeed, collectible card games like *Magic*: *The Gathering* have employed a similar mechanic for decades (a full review of the history of these ‘randomised reward mechanisms’ in games is available in [17]). This has resulted in industry pressure groups and representatives repeatedly equating the effects of loot boxes with these other forms of entertainment, which are themselves perceived to be harmless. For example, the ESRB have recently claimed that there is insufficient evidence that loot boxes had negative consequences for gamers [18]. They instead declared that “we do not consider loot boxes to be gambling for various reasons … loot boxes are more comparable to baseball cards, where there is an element of surprise and you always get something.” [18]. Similarly, The IGEA is the industry body responsible for representing the business and public policy interests of gaming companies in Australia and New Zealand. They liken loot boxes to harmless Kinder Surprise chocolates and state that “When you purchase a Kinder Surprise, you might receive a prize you already own or one that you do not. Loot boxes operate in the same way, as they too offer a variety of different items.”[19].

However, in contrast to these statements, recent data has suggested an important behavioural association between loot box spending and problem gambling. In [15], researchers found that purchasing loot boxes was associated with problem gambling in a sample of eSports spectators; in a recent large scale survey (n = 7,422) [20], researchers polled a group of gamers and found that the problem gambling of gamers had a significant (η^2^ = 0.054) relationship with their loot box spending.

It is important to point out that this research is potentially limited by the nature of the samples under test: In [15], eSports spectators rather than gamers were polling, limiting the generalisability of results. Similarly, in [20], gamers were recruited by asking whether they would like to take part in a survey on loot boxes and gambling. Whilst they may not have been aware of the specific aims of the study, they would certainly have been aware of the general issues it examined and would have self-selected into the survey on the basis of their interest in these issues. This may also limit the generalisability of this research–indeed, in the conclusions of both studies, authors explicitly called for further work to be conducted which addressed these limitations and confirmed the robustness of their findings.

The research that we present below directly addresses this gap in the literature. We conducted a similar survey to [21], and measured both gamers’ problem gambling, and their spending on loot boxes. However, crucially, in the research presented here, gamers did not self-select into a study about loot boxes; and any gamers who showed suspicion that the study itself might be about loot boxes and gambling were removed from our sample. Our results support the robustness of previous findings on the effects of loot boxes. More specifically they confirm the size and positive correlation of the relationship between loot box spending and problem gambling that was previously observed. This information is of direct and urgent relevance to ratings boards and gambling regulators.

## Method

This research was approved by the Cross-School Research Ethics Committee for the Schools of Art, Design & Computer Science; and Performance & Media Production at York St. John University

### Design

We conducted an online survey with a sample of gamers aged 18 or older. Participants were recruited via an advertisement on Amazon Mechanical Turk order to answer a survey about their spending habits in games. In contrast to previous research, the recruitment message specifically did not mention loot boxes. Instead it read “We are conducting a survey about the different things that gamers spend money on, and how much they spend on each of these things.”.

Participants were screened before beginning the survey to ensure that they regularly played one of the 10 most globally popular games that feature loot boxes: *Player Unknown’s Battlegrounds*, *League of Legends*, *Hearthstone*, *Overwatch*, *Counter-Strike*: *GO*, *FIFA 18*, *Rocket League*, *DOTA 2*, *Team Fortress 2*, and *Tom Clancy’s Rainbow Six Siege*. At the end of the study, for the purposes of screening, they were asked these questions again to ensure consistency in their responses

This study was designed to measure problem gambling and loot box spending in a sample of gamers from the USA. For extensibility to other studies, all measures of spending in this study relied on participants reporting spending in the currency of their home country. For example, if a participant listed their nationality as ‘Australian’ it would ask for their spending in Australian Dollars rather than USA Dollars. 2 participants that took part in the study were not from the USA, but rather from India and Australia. They therefore reported their spending in Indian rupees and Australian dollars respectively. However, both of these participants did not spend any money on either loot boxes or other microtransactions. Therefore, conversion into US dollars was not necessary as all measurements were essentially in US dollars already (i.e. 0 Indian rupees is the same as 0 US dollars, which is the same as 0 Australian dollars).

**Loot box spend** was measured by asking participants to state approximately how much money they had spent on loot boxes in the past month. In order to blind participants to the aims of the study, they were also asked a variety of other questions about their spending habits: How much money they spent on physical copies of video games; how much money they spent on virtual copies of video games; and how much money they spent on in-game items.

**Problem gambling** was measured using the Problem Gambling Severity Index (PGSI) [22]. The PGSI consists of a series of 9 questions which each ask the participant how frequently they engage in some behaviour that is related to problem gambling. For example, one question asks participants how often over the past month “Has your gambling caused any financial problems for you or your household?”. Each of these questions is answered on a 4-point scale, with the following scoring pattern: (0) Never; (1) Sometimes; (2) Most of the time; (3) Almost always. The sum of scores over all 9 questions gives a total PGSI score that ranges from 0 (i.e. all questions answered as ‘Never’) to 27 (i.e. all questions answered as ‘Almost always’).

Participants were presented with the 9 items from the PGSI within a larger series of questions which they were informed related to impulsiveness. Participants were then classified as either ‘non problem gamblers’ (Score: 0), ‘low-risk gamblers’ (Score: 1–4), ‘moderate-risk gamblers’ (Score: 5–7), or ‘problem gamblers’ (Score: 8+) using the revised scoring system for the PGSI [23]. This scoring scheme separates gamblers into classification bands on the basis of how extreme and frequent the problems are that their gambling has caused, rather than the absolute amount that they have spent. Thus, an individual who reports several gambling-related problems occurring frequently within their life might be classified as a problem gambler, whilst an individual who reported a lack of gambling-related problems would not, regardless of how much money each of these gamblers spent.

Each item was scored on a 4-point scale, giving a total score of 0–27.

At the conclusion of the survey, participants were asked what they thought the survey was about. Any participants who gave answers that contained both ‘loot boxes’ and ‘gambling’, or any variants of these words, were removed from the sample. This is in contrast to previous research, in which participants were aware that the study concerned both loot boxes and gambling.

The survey itself took an average time of 4 minutes and 38 seconds to complete. Participants were paid $0.60 for their time, equivalent to $7.80/hour.

### Participants

1,545 responses were collected in total from gamers from the USA. 245 respondents gave more than one inconsistent answer to the ten screening questions when they were repeated at the end of the study and were removed from the sample. 119 participants mentioned both loot boxes and gambling when asked what they thought the study was about and were removed from the sample. 7 participants listed their gender as a number. They were deemed non-serious and removed from the sample. This left a total of 1,174 responses. 1,172 participants listed their nationality as ‘United States’. One participant listed their nationality as Indian, and one participant listed their nationality as Australian. The intention with this study was to recruit gamers solely from the USA, and it was determined that these two gamers should be removed from the study, leaving a sample of 1,172 gamers.

751 participants (64%) described themselves as male. 372 participants (31%) described themselves as female. The remaining 50 participants (4%) gave other answers.

237 participants (20%) were aged 18–24. 342 (29%) were aged 25–29; 300 (25%) were aged 30–34; 148 (12%) were aged 35–39; 150 (12%) were aged 40 or over.

## Results

Reported spending on loot boxes over the past month ranged from $0 to $2300. Means and 95% confidence intervals of loot box spending when split by problem gambling severity are presented below as Table 1.

**Table 1:** Means and 95% confidence intervals for player spending on loot boxes, split by problem gambling severity.

	Loot box spend	N
Non problem gamblers	$11.14 (95% CI: $4.19 –$18.09)	596
Low-risk gamblers	$21.87 (95% CI: $7.10 –$36.64)	313
Moderate-risk gamblers	$27.55 (95% CI: $1.64 –$53.46)	56
Problem gamblers	$38.24 ($16.66 –$59.82)	207
Total	$19.58 (95%CI: $12.94 - $26.21)	1172

**Fig 1:**
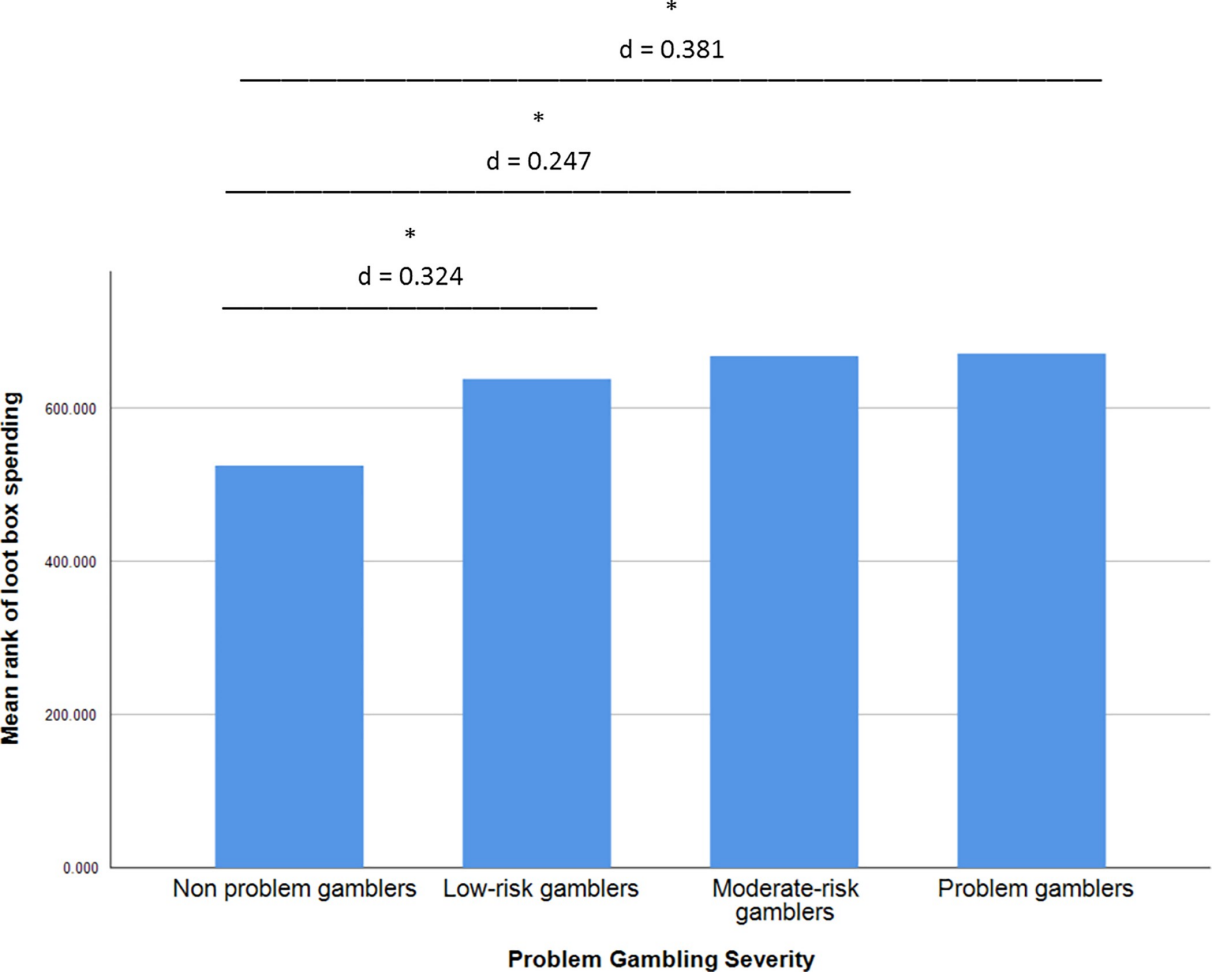
Barchart representing pairwise comparisons of the effects of problem gambling on loot box spending. Significant effects at the p<0.008 level are shown with lines, and annotated with effect sizes in Cohen's d.

The effects of problem gambling (non problem gamblers, low-risk gamblers, moderate-risk gamblers, problem gamblers) on loot box spend were tested via Kruskal Wallis H Test. In order to support the robust, diverse interpretation of tests, we present both the commonly used parametric measures of effect size, η^2^ and d, alongside the common language effects sizes of Vargha and Delaney (2000), namely Absolute Average Deviation (AAD) and stochastic superiority A. Results indicated that there was a statistically significant effect of problem gambling on loot box spending, χ^2^(3) = 62.850, p<0.001, η^2^ = 0.051 AAD = 0.081. Pairwise comparisons were then conducted via a series of 6 Mann-Whitney U tests. Bonferroni corrections were applied to the results of these tests, raising the alpha level of the tests to 0.05/6, or 0.008. The results of these comparisons are reported below as Table 2, and depicted visually as Fig 1.

**Table 2:** Pairwise comparisons of the effects of problem gambling on loot box spending.

Pairwise comparison groups	U	p-value	Cohen’s d	Vargha and Delaney’s A’
Non problem gamblers vs. low-risk gamblers	75140.00	<0.001*	0.429	0.597
Non problem gamblers vs. moderate-risk gamblers	12467.00	<0.001*	0.568	0.622
Non problem gamblers vs. problem gamblers	46432.00	<0.001*	0.548	0.623
Low-risk gamblers vs. moderate-risk gamblers	8228.00	0.416	0.102	0.526
Low-risk gamblers vs. problem gamblers	30287.00	0.163	0.121	0.531
Moderate-risk gamblers vs. problem gamblers	5756.50	0.932	0.023	0.506

Effects that are significant at the p<0.008 level are marked with a *.

### Exploratory analyses

In order to clarify whether the strength of the relationship between problem gambling and loot box spending observed above was specific to loot boxes, exploratory analyses were conducted on players’ responses to the question “During the last month, approximately how much money in dollars would you say that you have spent on in-game items per month? (Excluding loot boxes)”. As noted in the design subsection of our method, this question was initially asked solely as a way of blinding participants to the purpose of the study. Means and 95% confidence intervals of spending on in-game items when split by problem gambling severity are presented below as Table 3.

**Table 3:** Means and 95% confidence intervals for player spending on other in-game items, split by problem gambling severity.

	Spending on in-game items other than loot boxes	N
Non problem gamblers	$40.12 (95% CI: $-0.38 –$80.62)	596
Low-risk gamblers	$30.98 (95% CI: $11.49 –$50.48)	313
Moderate-risk gamblers	$36.07 (95% CI: $18.55 –$53.58)	56
Problem gamblers	$78.83 (95%CI: $19.59 - $138.07)	207
Total	$44.32 (95% CI: $20.67 - $67.98)	1172

The effects of problem gambling (non problem gamblers, low-risk gamblers, moderate-risk gamblers, problem gamblers) on spending on in-game items other than loot boxes were tested via Kruskal Wallis H Test. Results indicated that there was a statistically significant effect of problem gambling on spending on in-game items, though with what appeared to be a smaller effect size than the relationship between problem gambling and loot box spending observed above, χ^2^(3) = 32.470, p<0.001, η^2^ = 0.025, AAD = 0.071.

Exploratory pairwise comparisons were then conducted via a series of 6 Mann-Whitney U tests. Bonferroni corrections were applied to the results of these tests, raising the alpha level of the tests to 0.05/6, or 0.008. The results of these comparisons are reported below as Table 4.

**Table 4:** Pairwise comparisons of the effects of problem gambling on spending on in-game items other than loot boxes.

Pairwise comparison groups	U	p-value	Cohen’s d	Vargha and Delaney’s A
Non problem gamblers vs. low-risk gamblers	78292.00	<0.001*	0.300	0.580
Non problem gamblers vs. moderate-risk gamblers	11829.00	<0.001*	0.527	0.639
Non problem gamblers vs. problem gamblers	51755.00	<0.001*	0.298	0.579
Low-risk gamblers vs. moderate-risk gamblers	7592.00	0.099	0.217	0.561
Low-risk gamblers vs. problem gamblers	31646.00	0.642	0.036	0.510
Moderate-risk gamblers vs. problem gamblers	6364.00	0.243	-0.160	0.455

Effects that are significant at the p<0.008 level are marked with a *.

The Spearman rank correlation matrix for loot box spending, other microtransaction spending, and problem gambling categorisation was computed. This is shown below as Table 5.

**Table 5:** Correlation matrix for Spearman rank correlations between problem gambling classification, loot box spending, and other microtransaction spending.

	Problem gambling classification	Loot box spending	Other microtransaction spending
**Problem gambling classification**			
**Loot box spending**	0.228		
**Other microtransaction spending**	0.155	0.454	

All correlations are significant at the p<0.001 level.

The same analysis was then conducted on the relationship between raw problem gambling scores (i.e. scores ranging from 0 to 27) and the spending variables outlined above. The Spearman rank correlation matrix for these variables is displayed below as Table 6.

**Table 6:** Correlation matrix for Spearman rank correlations between raw problem gambling scores, loot box spending, and other microtransaction spending.

	Problem gambling classification	Loot box spending	Other microtransaction spending
**Problem gambling classification**			
**Loot box spending**	0.238		
**Other microtransaction spending**	0.164	0.454	

All correlations are significant at the p<0.001 level.

We then conducted a series of exploratory analyses to investigate whether the relationship between other microtransactions and problem gambling was significantly weaker than the relationship between loot box spending and problem gambling. In order to assess the difference between these relationships, they were entered into statistical analyses which allowed the testing of interactions between different kinds of spending and problem gambling severity.

A mixed-model ANOVA was first conducted, with problem gambling (non problem gamblers, low-risk gamblers, moderate-risk gamblers, problem gamblers) as a between-participants factor and type of spending (loot box spending, spending on in-game items other than loot boxes) as within-participants factor. Results gave no evidence for either an effect of problem gambling categorisation (F(3,1168) = 1.135, p = 0.333), a main effect of type of spending (F(1,1168) = 1.761, p = 0.184), or an interaction between these factors (F(3,1168) = 0.327, p = 0.805).

However, It is important to note that the data that are under test are non-normal with the data for both loot box and microtransactions spend exhibiting long tails in all categories of problem gambling: for example, whilst the mean spending on loot boxes was $19.58, this data ranged from $0 to $2300, indicating the presence of extreme outliers. A Shapiro-Wilk test for normality on each variable in each problem category supported this with W < 0.6 and p < 0.001 in all cases.

We therefore ran a follow-up nonparametric analysis whose assumptions did not require data to be normally distributed. More specifically, an Aligned Rank Transform test, analogous to a nonparametric ANOVA, was conducted according to [24], with problem gambling (non problem gamblers, low-risk gamblers, moderate-risk gamblers, problem gamblers) as a between-participants factor and type of spending (loot box spending, spending on in-game items other than loot boxes) as within-participants factor. Results indicated a significant effect of problem gambling categorisation (F(3,1168) = 18.68 p<0.001), a main effect of type of spending (F(1,1168) = 232.40, p<0.001), and an interaction between these factors (F(3,1168) = 46.80, p<0.001).

To confirm the presence of this effect, a robust 5% trimmed mixed-model ANOVA was conducted according to [25], with problem gambling (non problem gamblers, low-risk gamblers, moderate-risk gamblers, problem gamblers) as a between-participants factor and type of spending (loot box spending, spending on in-game items other than loot boxes) as within-participants factor. Results indicated a significant effect of problem gambling categorisation (F(3,1168) 12.28, p<0.001), a main effect of type of spending (F(1,168) = 19.60,p<0.001), but no interaction between these factors (F(3,1168) = 1.18, p = 0.319).

## Discussion

### Loot box spending is linked to problem gambling

The results of this study provide further evidence of a potentially important relationship between problem gambling and loot box spending. Overall, there was a significant link between participants’ scores on the Problem Gambling Severity Index and their loot box spending (p<0.001, η^2^ = 0.051). Individuals who did not have gambling problems spent significantly less money on loot boxes than those who were problem gamblers, or at risk of problem gambling.

Subgroup analyses revealed that on average, non problem gamblers spent significantly less money per month on loot boxes (mean = $11.14) than either low-risk gamblers (mean = $21.87), moderate-risk gamblers (mean = $27.55), or problem gamblers (mean = $38.24).

Not only does the direction of these effects tally with those seen in Zendle and Cairns’[20] previous investigation of the effects of loot boxes, but the effect sizes associated with these relationships align closely with this previous work too. In [20], an overall relationship between loot box spending and problem gambling was observed that was of magnitude η^2^ = 0.054. Here, the overall relationship we see is of magnitude η^2^ = 0.051. Zendle and Cairns’ subgroup analyses found differences between non-problem gamblers and other subgroups of magnitudes ranging from Cohen’s d = 0.277 to Cohen’s d = 0.368. Here, our subgroup analyses revealed ranges in effect size from Cohen’s d = 0.429 to Cohen’s d = 0.568.

However, when it comes to analysing differences between other subgroups, it is interesting to note that whilst differences were observed between non problem gamblers and both low-risk, moderate-risk, and problem gamblers, no significant differences were observed within these groups. Our results gave no evidence to support the idea that low risk, moderate risk, and problem gamblers differed from each other in terms of their spending on loot boxes, in contrast to [21]. There are several reasons why this might be the case. Firstly, it may be the case that real differences exist in the world, but our sample was not large enough to observe these effects. It is worth noting that in this dataset, for instance, only 56 moderate risk gamblers formed part of the sample.

It is possible that there are other explanations for this lack of an effect. Differences are thought to exist between individuals who are categorised as ether low-risk, moderate-risk, and problem gamblers. For example, problem gamblers have been observed to gamble more, and have higher levels of debt than moderate-risk gamblers[26]. However, as noted in [23], demographic and behavioural differences between low and moderate risk categories are often small and statistically insignificant. Indeed, in some studies, these classification categories are collapsed into each other (e.g. [27], [28]). Further studies are needed with larger numbers of gamers that fall into these categories in order to determine whether the lack of an effect observed here between these subgroups is simply a Type II error, or whether it really does represent a meaningful facet of the relationship between loot box spending and problem gambling amongst gamers.

It is important to note that the overall relationship between problem gambling and loot box spending that was observed is of small-to-medium size. This suggests that the relationship between problem gambling and loot box spending may be comparable in strength to the relationship between problem gambling and known risk factors in the gambling literature. For instance, the relationship between problem gambling and current alcohol dependence is estimated at approximately η^2^ = 0.0625 (equivalent Cohen’s d = 0.516) [29]. Interestingly, other factors that relate to technology use have been estimated to have similarly strong links with problem gambling. For example, exposure to gambling content in social media has been shown to share a moderate-strength relationship with problem gambling[30], as has engagement with simulated gambling in digital and social media [31]. It may therefore be the case that the links between loot boxes and problem gambling that we see here are not an isolated phenomenon. They may instead be a single indicator of a phenomenon, as new forms of communication and entertainment technology allow audiences easy access to novel gambling-like and gambling-related experiences.

### Other microtransaction spending and problem gambling

In [21], the relationship between problem gambling and other microtransaction use appeared trivially small (η^2^ = 0.004). However, a much stronger relationship was observed here, of magnitude η^2^ = 0.025. Though the picture of loot box spend is consistent, the complexity of the microtransaction spend indicates how important it is to gather more data in this area. Exploratory follow-up analyses painted an inconsistent picture: An aligned rank transform revealed a significant interaction between types of spending and problem gambling, indicating that the relationship between other microtransactions spending and problem gambling is inferior to the relationship between loot box spending and problem gambling. However, this effect was not consistently seen during exploratory analyses, and did not appear in either a mixed ANOVA, or a robust 5% trimmed ANOVA. Much more work is needed first to understand the structure of the data in order to determine which more sophisticated modelling approaches might be appropriate to its analysis. In particular, based on our data here we might propose a generalized linear model of the spend data that could then be used as the foundation for future studies and their analysis. This further opens up the opportunity for Bayesian analysis that can consider robust parameter estimation of such models. It would be premature to do such analysis on our current data because until this study, we had no conception of what the data might look like and therefore what might be suitable models.

### Limitations

An additional note must be made about the sample that was used in this study. An unusually large number of participants in this study identified as problem gamblers. Overall, 207 individuals from within our 1172 participant sample scored 8 or higher on the PGSI. This indicates a much higher level of problem gambling in our sample than in the population at large, in which problem gambling is relatively rare [23]. It seems likely that the prevalence of problem gamblers in our sample is due to the data collection method we employed: In this study, we recruited participants via Amazon Mechanical Turk, a popular microwork platform. It may be the case that microworkers are more likely to suffer from problem gambling than the general population. This point is supported by previous research on gambling amongst microworkers. For example, in [32], researchers examined the usefulness of the Crowdflower microwork platform for recruiting individuals with gambling problems. They found that as many as 24% of the participants that they recruited were problem gamblers. The data collection method we employed here may therefore have allowed us a good opportunity to study the behaviours of problem gamblers who are also gamers. Similarly, previous studies on loot boxes have seen a large gender imbalance amongst participants: In [15], only 5.5% of participants were female; in [21] only 9%. By contrast, in the sample used here, almost a third of participants identified as female. Again, the difference between these samples may be due to the data collection method employed. The fact that similar patterns of results were replicated in this study despite the different composition of the sample under test suggests that the link between problem gambling and loot box spending may generalise widely across different groups of gamers. Further work is necessary to confirm that this is the case.

Finally, it is key to note that the specific methodology followed during this study itself carries inherent limitations. In order to assess the level of gamers’ spending on loot boxes, we asked them to self-report how much they had spent on these things over the past month. It may be the case that these estimates are imprecise, and that taking a direct measure of actual spending would allow a more precise evaluation of the strength of any link between problem gambling and loot box spending. Additionally, this work is primarily concerned with the replication of previous work on links between the amount that individuals spend on loot boxes and their problem gambling severity. Therefore, it does not focus on factors such as the frequency of loot box opening, how long it takes gamers to open loot boxes, or individuals’ exposure to loot box opening videos on websites like YouTube. However, all of these factors may be of importance when it comes to any relationship between loot box spending and problem gambling.

## Conclusions

This research provides further evidence of a potentially important link between problem gambling and the amount that individuals spend on loot boxes. It directly addresses the limitations of previous research, in which a similar link was seen in an unblinded and self-selected sample. This research replicates that relationship and suggests that it remains in existence even when a sample is unaware of the fact that research concerns loot boxes and gambling, and have not self-selected into a loot box-related study.

However, it is key to note that the causal direction of this relationship is unclear. It may be the case that loot boxes cause individuals to become problem gamblers. It may also be the case that pre-existing gambling problems cause individuals to spend more money on loot boxes.

If this is the case, the presence of loot boxes in video games would not be creating a ‘breeding ground’ for problem gambling. They would instead be providing an opportunity for games companies to exploit serious pre-existing psychological problems amongst their customers for massive monetary gains. The correlational nature of this study makes it impossible to determine which of these pictures of the effects of loot boxes is true.

However, regardless of the direction of causality, the games industry faces a crisis of conscience. Industry bodies such as the ESRB and IGEA are finding it increasingly difficult to claim in good faith that there is little evidence of a link between problem gambling and loot box use. Loot boxes are a novel phenomenon, and game developers may understandably be wary of the association of their products with gambling. However, as noted in [33], in this case the “emphasis for all parties, be they government, industry, or consumer, should be on the need for self‐education and due diligence in understanding the complexity and nuance of games and gambling.”. We strongly believe that this encompasses the need for continual reflection on the potential effects of loot boxes on the behalf of industry stakeholders

It is our view based on the findings of this study that ratings agencies should consider incorporating additional parental advisories into games that persist in featuring loot boxes. In [34], King and Delfabbro outline a broad variety of different ways that loot box related harm may be mitigated by employing social responsibility measures. In light of the results seen here, we believe that many of the suggestions that are suggested in that document are appropriate. Most importantly, we follow them in their suggestion that appropriate content descriptors are added to games that feature loot boxes. We recommend that games with loot boxes are restricted to players of legal gambling age. It is also our opinion that the severity of the link seen here suggests that relevant authorities should seriously consider restricting access to loot boxes as if they were a form of gambling.
